# Systematic Review with Meta‐Analysis of Biofluid Markers for Huntington's Disease

**DOI:** 10.1002/mds.70067

**Published:** 2025-10-13

**Authors:** Jane S. Paulsen, Natalie A. Bovin, Jordan D. Clemsen, Alexander Weiss, Abigail M. Key, Monica L. Janz, Alex Pinto, Deven K. Burks, Henrik Zetterberg, Kathleen M. Shannon

**Affiliations:** ^1^ Department of Neurology University of Wisconsin School of Medicine and Public Health Madison Wisconsin USA; ^2^ Department of Neurology University of Colorado Anschutz Medical Campus Aurora Colorado USA; ^3^ Tri‐institutional Center for Translational Research in Neuroimaging and Data Science (TReNDS), Georgia State University, Georgia Institute of Technology, Emory University Atlanta Georgia USA; ^4^ Evotec SE Hamburg Germany; ^5^ Department of Biostatistics and Medical Informatics University of Wisconsin School of Medicine and Public Health Madison Wisconsin USA; ^6^ Department of Psychiatry and Neurochemistry Institute of Neuroscience and Physiology, the Sahlgrenska Academy at the University of Gothenburg Mölndal Sweden; ^7^ Clinical Neurochemistry Laboratory, Sahlgrenska University Hospital Mölndal Sweden; ^8^ Department of Neurodegenerative Disease UCL Institute of Neurology London UK; ^9^ UK Dementia Research Institute at University College London London UK; ^10^ Hong Kong Center for Neurodegenerative Diseases Hong Kong China; ^11^ Wisconsin Alzheimer's Disease Research Center, University of Wisconsin School of Medicine and Public Health Madison Wisconsin USA; ^12^ Centre for Brain Research, Indian Institute of Science Bangalore India

**Keywords:** biofluids, biomarkers, Huntington's disease, meta‐analysis, systematic review

## Abstract

Primary therapeutic objectives for Huntington's disease (HD) necessitate continued therapy for a long period before clinical motor diagnosis and its concurrent functional incapacities. Therefore, the need is paramount for alternative biomarkers that are not only highly sensitive but also clearly reflect the disease progression. Current trials increasingly rely upon biological definitions of disease to initiate intervention before significant decline. The primary biological measure of early‐stage HD is volumetric evidence of structural decline on magnetic resonance imaging. This comprehensive review of biofluid markers is a systematic review documenting 804 records identified in a literature search. Updating a previous comprehensive review from 2018, we summarize effect sizes and conduct meta‐analyses for 55 studies with reproducible findings. Evidence for neurofilament light (NfL) is sufficient to meet evidentiary guidelines as a prognostic biomarker in preHD (ie, before clinical motor diagnosis). Significant meta‐analyses are found for 24‐hydroxycholesterol (24‐OHC), 27‐hydroxycholesterol (27‐OHC), NfL, and T‐tau (total tau) in early‐stage HD and for cortisol, high‐density lipoprotein (HDL), mutant huntingtin (mHTT), and HTT in mid‐stage HD after clinical motor diagnosis. Despite over 800 published studies of biomarkers in HD and over 200 reviews of those efforts, the current state of the literature is limited by inconsistent reporting of necessary detail in existing reports. This is compounded by an inability to effectively compare outcomes and by continued publication when rigor is compromised, revealing a significant knowledge gap for HD clinical trial methodology improvements. Findings support validation for eight biofluid markers in HD: one in preHD, four in early‐stage HD, and four in mid‐stage HD after clinical motor diagnosis. © 2025 The Author(s). *Movement Disorders* published by Wiley Periodicals LLC on behalf of International Parkinson and Movement Disorder Society.

## Graphical abstract

Eight Huntington's Disease Biofluid Markers show Meta‐Analytic Significance Across HD‐ISS Stages. Meta‐analyses were conducted for two types of biomarkers: Different from demographically‐matched healthy controls and *Showing change over time.

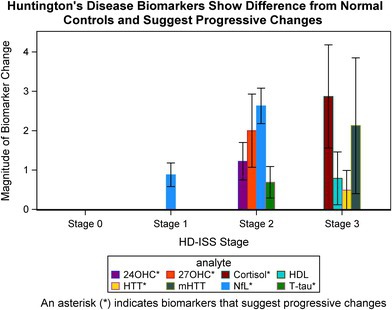

Huntington's disease (HD) is an autosomal dominant neurodegenerative disease with insidious worsening of cognitive, motor, and behavioral regulation over several decades. The causative gene for HD has cytosine‐adenine‐guanine (CAG) repeat expansion in exon 1 of the *HTT* gene on chromosome 4, which leads to a polyglutamine chain and codes for the huntingtin (HTT) protein.^1^ The biochemical cascade initiated by mutant *HTT* is complex and multifaceted, ultimately resulting in neuronal dysfunction and cell death. A pathological grading system for the postmortem brain based on the progressive loss of neostriatal spiny projection neurons and associated atrophy is established and has been observed in persons with HD even before clinical motor diagnosis.^2^ Clinical trials for HD started with the formation of the Huntington Study Group over 30 years ago. Early trials pursued improved clinical endpoints from the Unified Huntington's Disease Rating Scale (UHDRS), most often reducing chorea and maintaining functional capacity, culminating in U.S. Food and Drug Administration (FDA) approvals for tetrabenazine, valbenazine, and deutetrabenazine. Recent attention has focused on disease‐modification with attention to lowering the protein (ie, huntingtin [HTT]) produced by the mutant *HTT* gene through antibodies, cereblon E3 ligase modulatory drugs (CELMoDs), RNA‐ and DNA‐targeting therapies, antisense oligonucleotide (ASO), RNA splicing modulators, small molecules, adeno‐associated virus, zinc fingers, clustered regularly interspaced short palindromic repeats (CRISPR), and RNA interference (iRNA). Notably, the FDA granted regenerative medicine advanced therapy (RMAT) designation to one gene therapy trial on June 4, 2024 for their iRNA in HD following clinical motor diagnosis. Additional clinical trials recently reported positive results indicating potential disease modification.^3^ As potential HD therapeutics advance, the development and validation of biomarkers are vital.

Biomarkers, as defined by the FDA‐National Institutes of Health (NIH) working group, are “a defined characteristic that is measured as an indicator of normal biological processes, pathogenic processes, or biological responses to an exposure or intervention”.^4^ HD biomarker progress has accelerated even with limited documentation of representative data, universal standards, and validation processes. Consequently, vast literatures of biomarker investigations languish as markers are elevated by informal, unempirical methods. Despite apparent productivity of over 800 HD biofluid marker studies, conflicting findings exist, and many lack reproducibility.^5^, ^6^, ^7^, ^8^, ^9^ Presently, assays for HTT and neurofilament light (NfL) are used in HD clinical trials though no known marker has undergone evidentiary review for FDA qualification in any HD context of use. Widespread usage notwithstanding, the lack of formal qualification for these biomarkers may constrain or mislead therapeutic progress.^8^, ^10^ For example, HTT protein quantification assays require sensitive, novel, immune‐based technologies including fluorescence resonance energy transfer (TR‐FRET),^11^ meso scale discovery (MSD),^12^, ^13^ and single molecule counting (SMC).^14^, ^15^, ^16^, ^17^ Formal validation and widespread scalability are ongoing. Therefore, the absence of formal qualification may contribute to extraneous resource utilization and research participant burden. Since HTT is not detectable in over half of persons with HD before clinical motor diagnosis, efforts enrolling clinical trial participants at earlier stages of disease and prior to diagnosis operate in the blind. Recent findings using multiplex biomarker approaches suggest that, even with widely, repeatedly studied biomarkers (eg, NfL),^18^, ^19^, ^20^, ^21^ treatment strategies may require alternative or dual treatments to tackle complex, multidimensional pathologies observed in neurodegenerative diseases. Furthermore, since NfL is a general neurodegeneration biomarker detected in many neurological disorders, it is unknown how its transdiagnostic predictors of clinical outcomes interact with or synergize outcomes with condition‐specific proteomic biomarkers. Nevertheless, together with condition‐specific biomarkers, NfL holds promise as a biomarker for neurodegeneration intensity.

Though the FDA and NIH have clearly articulated guidance for the qualification of biomarkers, attention to standardization, harmonization, and prospective integration of promising biomarkers into clinical trial decision‐making remains incomplete. Biomarkers have several different uses in the drug development setting: diagnostic, monitoring, treatment response, predictive, prognostic, surrogate endpoint, safety, susceptibility, or risk.^4^ Publications increasingly highlight critical shortcomings in HD biomarker research that preclude necessary clinical trial improvements.^18^ Most biomarker publications in HD do not specify contexts of use though Tang and colleagues speculated that the majority are to monitor disease progression or predict a clinically relevant landmark (ie, prognostic biomarker).^20^ This systematic review aims to provide an update of HD biomarker advances building from previous reviews, summarize important developments, and formulate directions for future research. We speculate that improved tracking and consolidation of biomarker research findings will facilitate their translation so that clinical trials may be maximized by the extensive research being published.

## 1 Methods

**FIG. 1:**
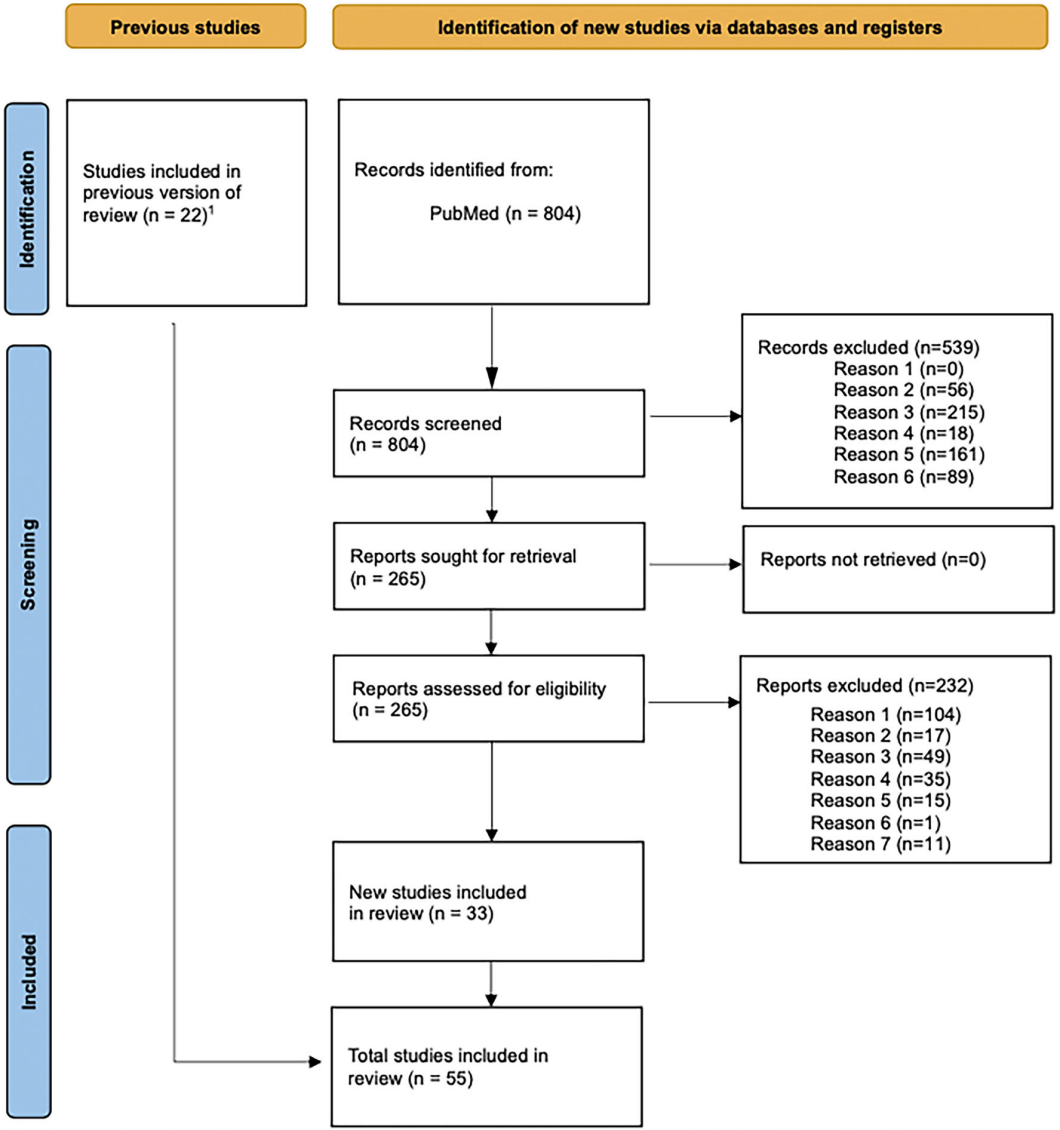
PRISMA (Preferred Reporting Items for Systematic reviews and Meta‐Analyses) flow chart for systematic review of biofluid markers in Huntington's disease (HD). *Source*: Page et al.^22^ This work is licensed under CC BY 4.0 To view a copy of this license, visit https://creativecommons.org/licenses/by/4.0/. ^1^Silajdzic and Bjorkqvist.^8^ [Color figure can be viewed at wileyonlinelibrary.com]

The following search terms were adapted from Silajdžić and Björkqvist's 2018 review: “Huntington's disease” or “Huntington disease” and “saliva” or “urine” or “blood” or “plasma” or “serum” or “cerebrospinal fluid” or “CSF.”^8^ To update from this previous review, a systematic literature search using PubMed and following PRISMA guidelines was performed from 02/18/2017 to 01/24/2025.^22^ Matching previous methods, an additional filter of English language articles was employed. The initial search yielded 804 articles, and a single reviewer reviewed abstracts to determine relevancy (N.A.B., with oversight from J.S.P. and M.L.J.). Some 265 articles met the review inclusion criteria, and full‐text study review and assessment of data quality were conducted by three independent reviewers (J.D.C., A.M.K., A.P.; Fig. 1). To meet the review inclusion criteria, articles were required to include persons with HD and normal controls (NC) and to have easily accessible data, either reported in the main article or any supplementary material. Consistent with biomarker guidelines, only analytes with replication were maintained in the final review and meta‐analysis (n = 33). Thus, any article that served as a replication of a single finding from Silajdžić and Björkqvist's 2018 review met the inclusion criteria for this review (n = 22) (see Table S1 for a description of codes used for exclusion from the study). Methodological quality of included publications was evaluated by two independent raters using the standardized 24‐item Biomarker Study Quality Assessment Tool for HD (BSQuAT‐HD).^20^

### 1.1 Statistical Analysis

Table S2 shows the publications detected since the previous review and interpreted in concert with Silajdžić and Björkqvist. For each analyte, sample sizes, means, and standard deviations were documented and analyzed via Hedge's standardized mean differences (SMD) between HD participants and NC. Hedge's g expresses the difference of the means in unit of pooled standard deviation and is visualized using forest plots. Random effects restricted maximum likelihood (REML) meta‐analysis was used to pool estimates of SMD for each biomarker and assess subgroup differences for analytes with replication. Multilevel meta‐regression models were fitted to account for correlated sampling errors where multiple effect sizes were nested within an individual study. The significance of pooled effect sizes was measured with the *t*‐test. All tests of statistical significance were two‐sided with *P* < 0.05 considered significant. The *I* ^2^ statistic was used to assess between‐study heterogeneity for each biomarker. Statistical analyses and data management were performed using SAS version 9.4 (SAS Institute, Cary, NC, USA) and R (version 4.2.0) in RStudio (version 2023.09.1 + 494). Like Silajdžić and Björkqvist, we divided the biomarkers into categories reflecting the previously used functional classifications. It is, however, important to note that such separations are somewhat artificial since pathways of neurodegeneration involve multiple intricacies of autophagy and mitophagy.^23^ Effect sizes (SMD with corresponding lower and upper confidence limits) are presented for each biomarker and study. When possible, findings are provided for distinct HD subgroups as color‐coded in the figures using abbreviations of preHD for participants before clinical motor diagnosis and HD with descriptors provided by authors (ie, early‐stage, mid‐stage, combined) for participants with HD after clinical motor diagnosis. This terminological choice reflects the variety of naming conventions from the existing literature and does not always follow recent guidance regarding preferred terminology and disease stages.^24^, ^25^

## 2 Results

### 2.1 Immune Markers

**FIG. 2:**
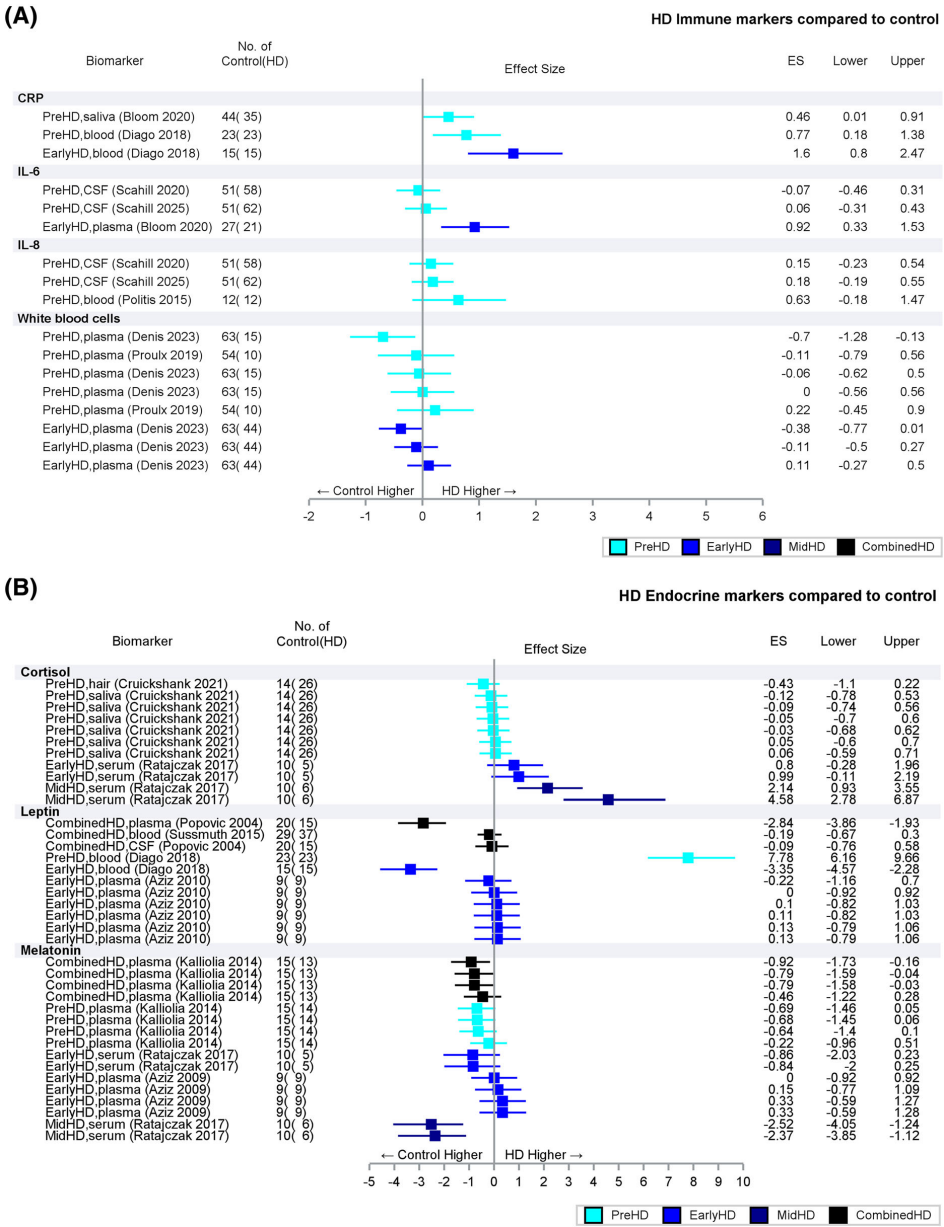
Effect sizes of immune markers and endocrine markers in Huntington's disease (HD). [Color figure can be viewed at wileyonlinelibrary.com]

New publications replicated increases in C‐reactive protein (CRP) in two more studies. Interleukin‐6 (IL‐6) added one increased finding after diagnosis and no difference in two additional studies before diagnosis. IL‐8 was elevated in one more study and nonsignificant in two. Effect sizes are illustrated in Figure 2A and show that the strongest immune marker effect size to date is CRP in early‐stage HD after clinical motor diagnosis with +1.6 effect size.

### 2.2 Endocrine Markers

The most frequently examined hormone since the previous review has been leptin though findings are mixed, with four suggesting no difference from NC. One study detected extreme effect sizes and interpreted findings as support for autonomic dysfunction (Fig. 2B) varying between persons before and after clinical motor diagnosis.

### 2.3 Metabolic Markers

**FIG. 3:**
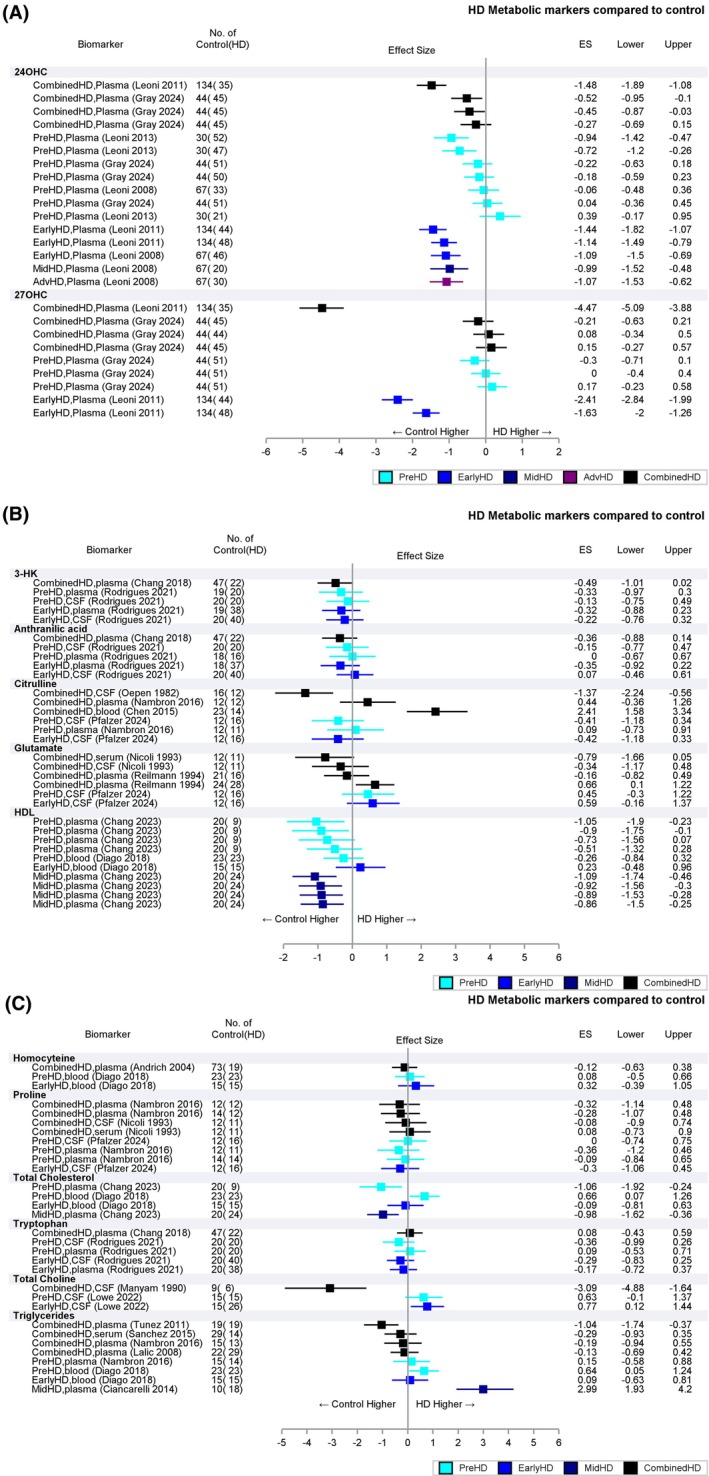
Effect sizes of metabolic markers in Huntington's disease (HD). [Color figure can be viewed at wileyonlinelibrary.com]

Metabolic markers were investigated more frequently than any other class in both the 2018 and current reviews (Fig. 3A–C), showing very large variations in effect sizes from −4.47 in 27‐hydroxycholesterol (27‐OHC) to 2.99 in triglycerides. 24‐Hydroxycholesterol (24‐OHC) showed decreases after clinical motor diagnosis across studies, although only one study showed earlier decreases before diagnosis. Across analytes, findings are heterogeneous, with assay variation limiting interpretation rigor. Consistent associations are reported between clinical phenotype and metabolic markers.

### 2.4 Oxidative Stress Markers

**FIG. 4:**
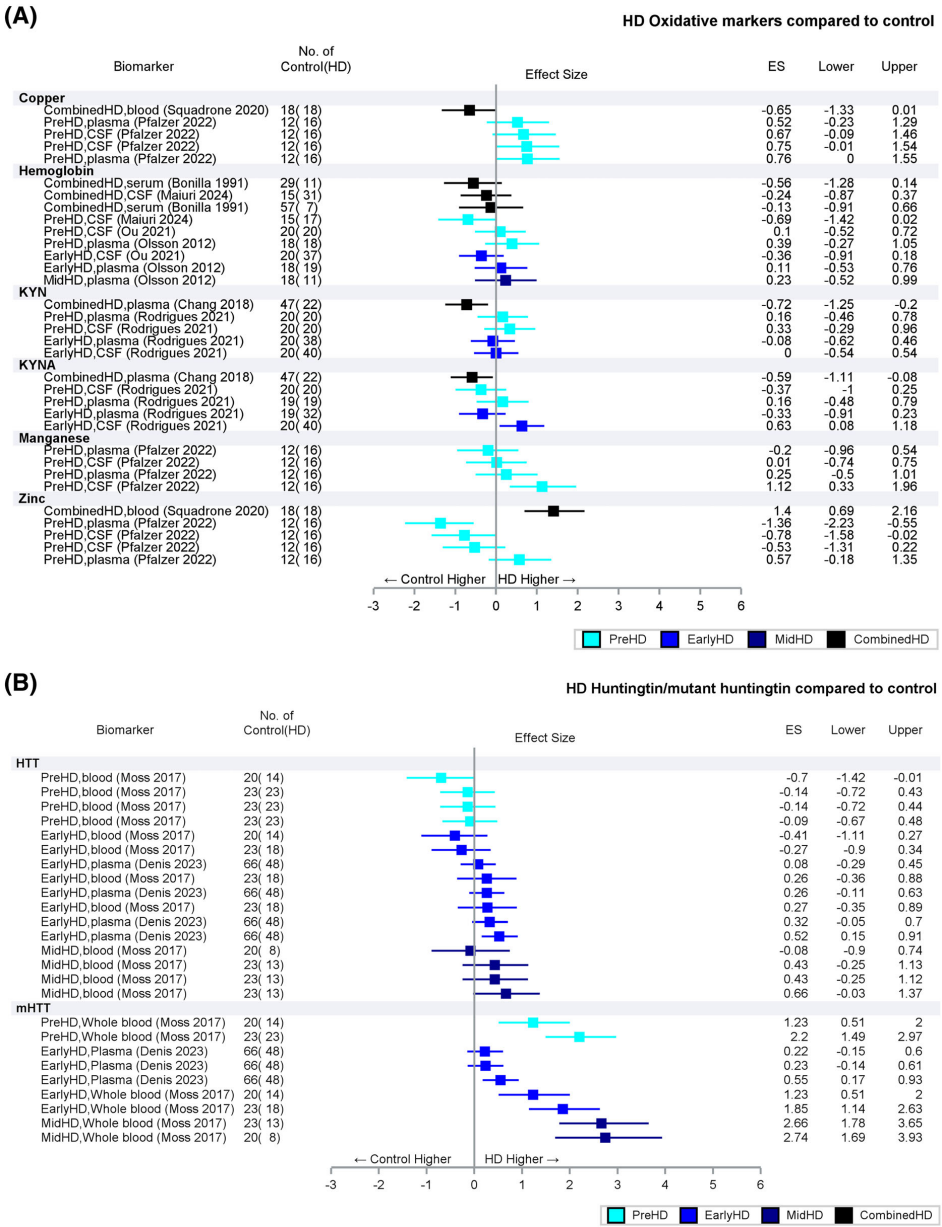
Effect sizes of oxidative markers and total and mutant huntingtin markers in Huntington's disease (HD). [Color figure can be viewed at wileyonlinelibrary.com]

The largest effect sizes in this group are manganese before (−1.36) and zinc after (1.4) clinical motor diagnosis (Fig. 4A), but biomarkers fail to display a clear pattern.

### 2.5 Total and Mutant HTT

Findings from this review show general consistency in direction and progression across the disease spectrum for HTT though studies lack consistent progression from before (−0.7) to after (up to 2.74 in mid‐stage HD) clinical motor diagnostic stages (Fig. 4B).

### 2.6 Axonal and Glial Degeneration

**FIG. 5:**
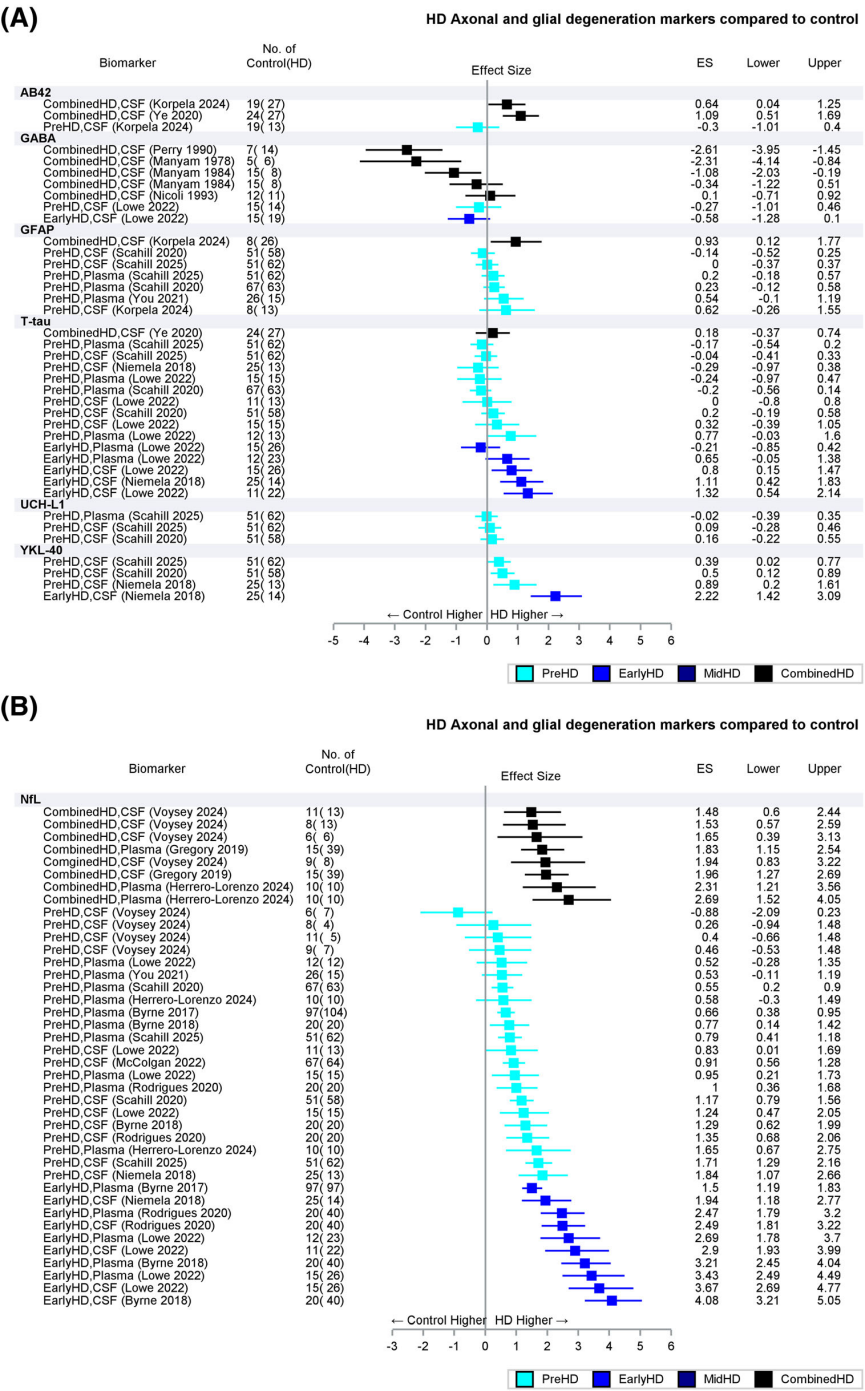
Effect sizes of axonal and glial markers in Huntington's disease (HD). [Color figure can be viewed at wileyonlinelibrary.com]

Figure 5A,B shows a clear progression of NfL across the disease spectrum for nearly every study with effect sizes from 1 to 2 before clinical motor diagnosis, followed by effect sizes over 4 after clinical motor diagnosis. While there are fewer findings and the effect sizes are not as robust, the patterns for glial fibrillary acidic protein (GFAP), total tau (T‐tau), and YKL‐40 similarly increase with disease progression.

## 3 Meta‐Analyses

Meta‐analyses are shown for each possible analyte in Table 1. We first conducted a meta‐analysis combining all HD participants regardless of reported HD staging as described (ie, HD vs. NC). Using this approach, NfL was highly significant with confidence levels from 1.16 to 1.88 and an *I* ^2^ of 89.3%. 24‐OHC was similarly robust, with confidence levels from −1.22 to −0.20 and an *I* ^2^ of 86.2%.

**TABLE 1:** Meta‐analysis of markers for any Huntington's disease (HD) and HD subgroups

Marker	Pooled effect estimate (g) (95% CI^a^)	*P*‐value^b^	Total *I* ^2^ ^c^ (%)	Articles (n) (effect sizes)^d^
Any HD				
Immune markers				
CRP	0.85 (−0.57, 2.27)	0.12	69.4	2 articles (n = 2 PreHD, n = 1 EarlyHD)
IL‐6	0.26 (−0.98, 1.50)	0.46	86.9	3 articles (n = 2 PreHD, n = 1 EarlyHD)
IL‐8	0.21 (−0.34, 0.76)	0.24	0.0	3 articles (n = 3 PreHD)
White blood cells	−0.14 (−0.37, 0.09)	0.20	16.7	2 articles (n = 5 PreHD, n = 3 EarlyHD)
Metabolic markers				
24‐OHC	−0.71 (−1.22, −0.20)	0.01	86.2	4 articles (n = 4 Combined, n = 7 PreHD, n = 3 EarlyHD, n = 1 MidHD, n = 1 AdvHD)
27‐OHC	−1.38 (−4.58, 1.81)	0.35	98.9	2 articles (n = 4 Combined, n = 3 PreHD, n = 2 Early HD)
3‐HK	−0.31 (−0.66, 0.04)	0.07	0.0	2 articles (n = 1 Combined, n = 2 PreHD, n = 2 EarlyHD)
Anthranilic acid	−0.17 (−0.53, 0.19)	0.25	0.0	2 articles (n = 1 Combined, n = 2 PreHD, n = 2 EarlyHD)
Citrulline	0.21 (−1.81, 2.24)	0.80	93.7	4 articles (n = 3 Combined, n = 2 PreHD, n = 1 EarlyHD)
Glutamate	0.10 (−0.71, 0.91)	0.77	67.6	3 articles (n = 4 Combined, n = 1 PreHD, n = 1 EarlyHD)
HDL	−0.50 (−1.42, 0.43)	0.26	71.8	2 articles (n = 5 PreHD, n = 1 EarlyHD, n = 4 MidHD)
Homocyteine	0.05 (−0.68, 0.77)	0.81	0.0	2 articles (n = 1 Combined, n = 1 PreHD, n = 1 EarlyHD)
Proline	−0.17 (−0.49, 0.16)	0.26	0.0	3 articles (n = 4 Combined, n = 3 PreHD, n = 1 EarlyHD)
Total cholesterol	−0.34 (−2.47, 1.80)	0.65	88.2	2 articles (n = 2 PreHD, n = 1 EarlyHD, n = 1 MidHD)
Total choline	−1.12 (−9.29, 7.04)	0.61	97.4	2 articles (n = 1 Combined, n = 1 PreHD, n = 1 EarlyHD)
Triglycerides	0.27 (−0.97, 1.51)	0.62	92.9	6 articles (n = 4 Combined, n = 2 PreHD, n = 1 EarlyHD, n = 1 MidHD)
Tryptophan	−0.12 (−0.47, 0.23)	0.38	0.0	2 articles (n = 1 Combined, n = 2 PreHD, n = 2 EarlyHD)
Endocrine markers				
Cortisol	0.73 (−1.14, 2.60)	0.40	90.2	2 articles (n = 7 PreHD, n = 2 EarlyHD, n = 2 MidHD)
Leptin	0.10 (−1.71, 1.90)	0.91	97.5	4 articles (n = 3 Combined, n = 1 PreHD, n = 7 EarlyHD)
Melatonin	−0.63 (−1.65, 0.39)	0.21	77.6	3 articles (n = 4 Combined, n = 4 PreHD, n = 6 EarlyHD, n = 2 MidHD)
Oxidative stress markers				
Copper	0.04 (−1.80, 1.88)	0.95	85.3	2 articles (n = 1 Combined, n = 4 PreHD)
Hemoglobin	−0.16 (−0.52, 0.21)	0.36	85.3	4 articles (n = 3 Combined, n = 3 PreHD, n = 2 EarlyHD, n = 1 MidHD)
KYN	−0.29 (−1.40, 0.82)	0.51	77.5	2 articles (n = 1 Combined, n = 2 PreHD, n = 2 EarlyHD)
KYNA	−0.15 (−0.94, 0.63)	0.62	70.8	2 articles (n = 1 Combined, n = 2 PreHD, n = 2 EarlyHD)
Zinc	0.32 (−2.32, 2.96)	0.75	93.0	2 articles (n = 1 Combined, n = 4 PreHD)
Axonal markers				
Aβ42	0.53 (−1.35, 2.42)	0.35	80.6	2 articles (n = 2 Combined, n = 1 PreHD)
GABA	−1.07 (−2.31, 0.17)	0.08	84.6	5 articles (n = 5 Combined, n = 1 PreHD, N = 1 EarlyHD)
GFAP	0.27 (−0.11, 0.65)	0.14	51.1	4 articles (n = 1 Combined, n = 6 PreHD)
NfL	1.52 (1.16, 1.88)	<0.0001	89.3	12 articles (n = 8 Combined, n = 22 PreHD, n = 10 EarlyHD)
T‐tau	0.22 (−0.06, 0.50)	0.12	65.6	5 articles (n = 1 Combined, n = 9 PreHD, n = 5 EarlyHD)
UCH‐L1	0.07 (−0.40, 0.55)	0.57	0.0	2 articles (n = 3 PreHD)
YKL‐40	0.91 (−0.37, 2.20)	0.11	89.1	3 articles (n = 3 PreHD, n = 1 Early HD)
Total and mutant huntingtin markers				
HTT	0.15 (−0.15, 0.45)	0.30	33.8	2 articles (n = 4 PreHD, n = 8 EarlyHD, n = 4 MidHD)
mHTT	1.11 (−0.69, 2.91)	0.19	94.0	2 articles (n = 2 PreHD, n = 5 EarlyHD, n = 2 MidHD)
HD subgroups				
CRP				
PreHD	0.57 (−1.73, 2.88)	0.20	0.0	2 articles (n = 2 PreHD)
IL‐6				
PreHD	−0.00 (−1.72, 1.72)	0.99	0.0	2 articles (n = 2 PreHD)
IL‐8				
PreHD	0.21 (−0.34, 0.76)	0.24	0.0	3 articles (n = 3 PreHD)
White blood cells				
PreHD	−0.15 (−0.52, 0.23)	(ref)	28.5	2 articles (n = 5 PreHD, n = 3 EarlyHD)
EarlyHD	−0.13 (−0.48, 0.23)	0.92		
24‐OHC				
PreHD	−0.25 (−0.57, 0.08)	(ref)	66.7	4 articles (n = 7 PreHD, n = 3 EarlyHD)
EarlyHD	−1.22 (−1.70, −0.75)	0.004		
27‐OHC				
PreHD	−0.04 (−0.84, 0.75)	(ref)	73.5	2 articles (n = 3 PreHD, n = 2 EarlyHD)
EarlyHD	−2.00 (−2.93, −1.07)	0.015		
Citrulline				
PreHD	−0.18 (−3.66, 3.31)	0.63	0.0	2 articles (n = 2 PreHD)
HDL				
PreHD	−0.57 (−1.19, 0.05)	(ref)	39.8	2 articles (n = 5 PreHD, n = 4 MidHD)
MidHD	−0.79 (−1.46, −0.12)	0.39		
Proline				
PreHD	−0.14 (−1.08, 0.81)	0.60	0.0	2 articles (n = 3 PreHD)
Total cholesterol				
PreHD	−0.18 (−11.10, 10.75)	0.88	91.2	2 articles (n = 2 PreHD)
Triglycerides				
PreHD	0.44 (−2.63, 3.50)	0.32	8.67	2 articles (n = 2 PreHD)
Cortisol				
PreHD	−0.09 (−0.69, 0.52)	(ref)	26.5	2 articles (n = 7 PreHD, n = 2 EarlyHD, n = 2 MidHD)
EarlyHD	0.89 (−0.14, 1.92)	0.09		
MidHD	2.87 (1.56, 4.18)	<0.01		
Leptin				
EarlyHD	−0.41 (−1.55, 0.72)	0.41	85.7	2 articles (n = 7 EarlyHD)
Melatonin				
EarlyHD	−0.08 (−0.60, 0.44)	0.72	7.8	2 articles (n = 6 EarlyHD)
Hemoglobin				
PreHD	−0.05 (−1.05, 0.95)	(ref)	65.3	3 articles (n = 3 PreHD, n = 2 EarlyHD)
EarlyHD	−0.33 (−1.43, 0.77)	0.42		
GFAP				
PreHD	0.14 (−0.09, 0.36)	0.18	0.0	4 articles (n = 6 PreHD)
NfL				
PreHD	0.88 (0.58, 1.18)	(ref)	74.5	11 articles (n = 22 PreHD, n = 10 EarlyHD)
EarlyHD	2.63 (2.18, 3.08)	<0.0001		
T‐tau				
PreHD	−0.00 (−0.26, 0.25)	(ref)	39.8	4 articles (n = 9 PreHD, n = 5 EarlyHD)
EarlyHD	0.69 (0.29, 1.09)	<0.01		
UCH‐L1				
PreHD	0.07 (−0.40, 0.55)	0.57	0.0	2 articles (n = 3 PreHD)
YKL‐40				
PreHD	0.50 (−0.04, 1.05)	0.06	0.0	3 articles (n = 3 PreHD)
HTT				
PreHD	−0.13 (−0.58, 0.33)	(ref)	30.4	2 articles (n = 4 PreHD, n = 8 EarlyHD, n = 4 MidHD)
EarlyHD	0.16 (−0.17, 0.50)	0.18		
MidHD	0.49 (−0.01. 0.99)	<0.05		
mHTT				
PreHD	1.15 (−0.47, 2.77)	(ref)	89.6	2 articles (n = 2 PreHD, n = 5 EarlyHD, n = 2 MidHD)
EarlyHD	0.91 (−0.57, 2.39)	0.56		
MidHD	2.13 (0.40, 3.85)	0.08		

Abbreviations: CI, confidence interval; HD, Huntingdon's disease; CRP, C‐reactive protein; IL, interleukin; 24‐OHC, 24‐hydroxycholesterol; 27‐OHC, 27‐hydroxycholesterol; 3‐HK, 3‐hydroxykynurenine; HDL, high‐density lipoprotein; KYN, kynurenine; KYNA, kynurenic acid; GABA, gamma‐aminobutyric acid; GFAP, glial fibrillary acidic protein; NfL, neurofilament light; T‐tau, total tau; UCH‐L1, ubiquitin carboxy‐terminal hydrolase L1; YKL‐40, chitinase‐3‐like protein 1; HTT, huntingtin; mHTT, mutant huntingtin; ref, reference.

^a^ Hedges’ g comparing (a) individuals with HD (any stage) to healthy controls or (b) each HD stage to healthy controls. A 95% CI including zero suggests no significant difference.

^b^ *P*‐value: Tests whether the pooled effect size differs significantly from (a) zero (i.e., whether HD is associated with a change in the biomarker compared to controls) or (b) preHD (e.g. early‐stage HD shows no difference in white blood cells (*P* = 0.92) while mid‐stage HD differs in cortisol (p < 0.01)).

^c^ Proportion of variability across studies due to heterogeneity rather than chance. Interpretation is limited for biomarkers with few studies.

^d^ Number of studies and total effect sizes included per biomarker.

Next, meta‐analyses were conducted for all cross‐sectional analytes among HD subgroups to determine whether longitudinal data would be meaningful to obtain for potential markers of disease monitoring. Only NfL shows both significance from normal and significant differences before clinical motor diagnosis. Many biomarkers showed meta‐analytic differences before versus after clinical motor diagnosis with significant differences of early‐ or mid‐stage HD from preHD. NfL had slightly higher confidence intervals of 2.18 to 3.08 for participants in early stages after clinical motor diagnosis. T‐tau was also significant after clinical motor diagnosis with confidence intervals of 0.29 to 1.09, as were 27‐OHC and 24‐OHC with confidence levels of −2.93 to −1.07 and −1.70 to −0.75, respectively. Huntingtin (HTT and mHTT), high‐density lipoprotein (HDL), and cortisol were significant after clinical motor diagnosis as mid‐stage HD biomarkers, with cortisol and mHTT having large effect sizes.

## 4 Methodological Rigor

Ratings of methodological rigor varied widely, with a notable lack of cross‐study consensus for many biomarkers. Overall, the mean BSQuAT‐HD score for included studies was 13.5 of 24 points. The most consistently reported methods included making a clear biomarker primary aim, providing a rationale for the study, reporting a prospective study design, providing confidence intervals, and discussing clinical applicability for the biomarker (ie, items 1, 2, 5, 21, and 24). Most (80%) reported clinical and demographic baseline characteristics and provided estimates of the biomarker's validity/reliability (items 18 and 23). Over 70% used linear mixed modeling instead of simple correlations and reported results in disease severity function. Only 60% of publications provided clear inclusion and exclusion criteria, 20% reported whether biomarker measurement was blinded, 17% addressed statistical power, and less than 12% included longitudinal biomarker acquisition. Few papers reported methodological handling of indeterminate, missing, or outlier data. Adverse events for biomarker collection, reliability or reproducibility, and potential sampling biases were rarely addressed.

## 5 Discussion

Conducted 7 years after another comprehensive review, this systematic review and meta‐analysis assessed biofluid markers in HD. Findings suggest eight biomarkers that demonstrate consistency across studies and rigor to maintain significance in meta‐analyses: NfL, 24‐OHC, 27‐OHC, T‐tau, cortisol, HDL, mHTT, and HTT. Substantial advances notwithstanding, this review's primary conclusion mirrors the 2018 review: biomarker discovery and validation in HD require improved methodological rigor.^8^ Despite over 800 reports of biofluid markers in HD, reproducibility is strikingly weak. One obvious explanation for the inconsistency may be secondary to assay variations across studies, but there are discrepancies across studies in HD sample selection and description that could constrain clear, transparent, generalizable reports.

This study's meta‐analytic findings show differences among HD subgroups. Since the publication of the first prognostic index combining CAG repeat length with current age,^26^ the field has witnessed an exponential leap forward in disease identification. Rather than staging persons with HD by phenotype or functional capacity, contemporary research utilizes one of several prognostic indices, alternatively called disease burden, CAG‐Age‐Product (CAP) score, or the prognostic index. Though the field heartily embraced this paradigm shift, variations in formula choice have limited clear, transparent cross‐study comparisons. Future biomarker studies could benefit from recommendations to standardize nomenclature so that all differences between HD groups on a specific biomarker are governed by the same disease progression metric.

Relatedly, although the current review acknowledges recent staging and nomenclature standards, many included publications did not observe these.^24^, ^25^ We have therefore integrated with these standards several preexisting terms that are progressively being phased out: preHD with “before clinical motor diagnosis” and HD stages with “after clinical motor diagnosis.” Arbitrary usage of before and after diagnosis, premanifest and early‐ versus mid‐stage HD, for example, needs to be replaced with the best CAG‐age‐product algorithm. This may facilitate more streamlined comparison of biomarker “a” with biomarker “b.” This recommended standardization could enable a biomarker timeline like that estimated in the Table of Contents Graphic using the HD Integrated Staging System (HD‐ISS).^25^

A prominent marker sensitive in many neurological diseases, NfL is a subunit of neurofilaments making up the neuronal cytoskeleton.^21^ Without contest, NfL is the most consistently significant biomarker currently available for HD. Briefly, NfL is correlated with the HTT levels,^17^, ^27^, ^28^, ^29^, ^30^, ^31^, ^32^, ^33^, ^34^ baseline motor and cognitive deficits, magnetic resonance imaging (MRI) caudate and putamen volumes,^35^ HD‐ISS stage,^25^ and Prognostic Index.^36^, ^37^ Notably, NfL predicts outcomes at 3 years, including rates of cortical thinning, white and gray matter volume loss, and cognitive, motor, and functional declines; it is more accurate than mHTT in the discrimination of cases before versus after clinical motor diagnosis.^29^ Though its biomarker relevance across neurological diseases is well‐established, FDA qualification is needed to document the specific assay and the contexts of use for HD. For instance, whereas many papers have replicated NfL as an HD prognostic marker, a recent publication suggests a prospective linear increase over time, implying usefulness in disease monitoring.^37^ This same group proposed NfL as a clinical trial sample enrichment method (exclude NfL < 45.01 pg/ml) to avoid including participants who are far from onset and in whom change may be more difficult to detect.^36^

There are, however, unresolved concerns in the widespread application of NFL in HD clinical trials, such as mixed findings of the consistency between plasma and cerebrospinal fluid (CSF): one recent longitudinal study reported excellent consistency^38^ while another specifically called out the inferiority of plasma NfL in disease monitoring.^39^ Correlations with other prognostic indicators show mixed findings across studies, with NfL failing to correlate in manifest disease, that is, associated with estimated years to onset before diagnosis but not associated with CAP score after diagnosis^36^ (cf. Scahill and colleagues' report).^40^ Most important are concerns about the lack of normative values for younger‐onset diseases such that early‐onset Alzheimer's disease failed to show differences from cognitively unimpaired normal controls when the fundamental impact of age‐related changes was not considered. Ashton and colleagues suggest that NfL cutoffs for persons aged less than 65 years were substantially lower compared with other cutoffs in neurodegenerative disease.^41^ Efforts in the literature to rectify inconsistencies in NfL measurement are ongoing, and the field needs better data. Despite massive advancements since the regulatory debut of NfL,^42^ extreme caution is advised for fieldwide precision and normative values of each specific assay, particularly considering recent NfL elevations in major depression, psychosis, bipolar, and substance use disorders.^43^ It may be prudent to adhere to one author's conclusion: “I wouldn't want to throw out a drug that otherwise looked promising because it didn't lower neurofilament levels”.^42^ Further research is urgently needed for HD to better detect and monitor progression in younger‐onset persons below the age of 65 years. One additional caveat with NfL as a biomarker for neurodegeneration is that its clearance seems to be mediated, at least in part, by microglia; hence, drugs that affect microglial activity may influence biofluid NfL concentrations.^44^

The HD community has devoted significant research resources to the development and refinement of assays for HTT for over a decade. Measures were developed in concert with clinical trials for gene therapies; these protein measures remain the primary target for treatment‐induced reduction. Studies documenting the reliability and validity of these markers are now available in CSF and blood. Given that HTT is typically undetectable in persons with HD before clinical motor diagnosis who are further from estimated onset, efforts to enhance assay sensitivities are ongoing.

In general, three types of biomarker assays are of interest for HTT‐lowering therapies: those measuring mHTT, total HTT, and healthy/wild‐type HTT. The latter is relevant because most HD carriers are heterozygous. The choice of analyte and sample type depends on the therapeutic approach. Systemic interventions such as orally provided small‐molecule splicing regulators that lower all HTT isoforms can often be monitored in blood‐based assays, which are readily accessible.^45^, ^46^ HTT is quite detectable in whole blood and peripheral blood mononuclear cells (PBMCs), using established immunoassays such as MSD, whereas measurements in plasma, serum, or CSF require high‐sensitivity platforms assays (eg, on the SMC).^17^, ^47^ For therapies targeting the central nervous system (CNS) and delivered intrathecally^48^, ^49^, ^50^, ^51^ or administered striatally,^52^ reductions in peripheral HTT are expected to be modest, making CSF the preferred matrix for monitoring target engagement. In CSF, HTT concentrations are very low (particularly before clinical motor diagnosis) and are quantifiable only with ultrasensitive assays.^16^, ^17^ Tear fluid, as an alternative matrix, shows surprisingly high mHTT levels and might be suitable for monitoring.^53^ The decision to measure mHTT versus total HTT depends on the drug's specificity. Allele‐selective approaches aim to reduce only mHTT while non‐selective strategies lower both forms. As mutant and wild‐type HTT differ only by polyglutamine length, no antibodies currently detect wild‐type HTT exclusively. Indirect quantification is possible by first depleting mHTT with polyQ‐targeting antibodies, then measuring the remaining protein with a total HTT assay.^15^

Further validation studies might be helpful to facilitate interpretations across platforms for clinical and research usage. As ongoing clinical trial data become available, the utility of these measures can be clarified with more comprehensive analyses. Publications in the current literature fail, however, to provide a consistent interpretation of the HTT outcomes. It must be noted that measures of HTT in late‐stage HD have not been reported and are needed to complete our understanding of the course of this biomarker. Other markers of axonal and glial degeneration in HD continue to be reported, leading to a profile of glial‐related inflammatory CSF biomarkers (eg, YKL‐40, GFAP) as well as cytoskeletal and myelin markers of neurodegeneration (eg, NfL, tau). A recent longitudinal study validates these cross‐sectional findings.^37^ Similar consistency is seen for tau in a review article, which confirms this observation.^54^ Rigor in future publications for these markers must be elevated for any publication to add to the literature. Further publications of nonstandard assays, unclear methods, and heterogeneous HD cases will fail to elevate the knowledge base for the discovery and validation of new axonal and glial markers in HD.

A wide range of cellular dysregulation is considered secondary to the HD gene mutation, including transcriptional dysregulation, mHTT protein aggregation, abnormal synaptic transmission, defects in cellular trafficking, proteostasis, and energy metabolism.^55^ Increasing evidence points to the involvement of immune system dysfunction in HD pathogenesis. Overproduction of reactive oxygen species (ROS) results in damage to nucleic acids, proteins, and lipids; oxidative stress from the mitochondria is a major source of elevated levels of ROS over a normal homeostatic state.^56^, ^57^, ^58^ Pro‐inflammatory cytokines have been reported as elevated in HD and other neurodegenerative diseases, even long before clinical motor diagnosis, indicating the importance of inflammatory states. Key components of defensive mechanisms are the nuclear factor kappa B protein complexes that regulate cellular responses to stimuli like stress, cytokines, and free radicals. The nuclear factor NFkB pathway acts as a transcription factor controlling the expression of genes involved in various cellular processes including inflammation, immunity, cell growth, and apoptosis.

Though the interactions within inflammasomes are poorly understood,^59^, ^60^ they have become a target for intervention in autoimmune diseases, inflammatory disorders, cancer, and neurodegeneration.^61^, ^62^ It may be noteworthy that HTT‐lowering reverses HD immune dysfunction caused by NFkB pathway dysregulation.^63^ Markers of axonal and glial degeneration in HD show consistent elevations over the HD course, leading to a profile of glial‐related inflammatory CSF biomarkers (eg, YKL‐40, GFAP, tau). A general immune marker, C‐reactive protein (CRP), consistently elevated in HD, has been shown to interact with antipsychotics in HD,^64^ so better controlled research is required to carefully assess CRP levels in patients while controlling for interactions with treatments. Reductions in white blood cells complement this finding, and inflammatory markers with signal differences cannot yet be ruled out given the pattern and consistency noted in the studies shown. Given the importance of the inflammasome in neuroimaging and in response to experimental therapeutics, further research should better characterize the relationships among immune system dysfunction and relevant phenotype and biomarkers in HD.

Over 60 studies have investigated endocrine markers in HD, but findings are challenging to interpret due to circadian rhythmicity, potential confounds, varying covariates, and methodological differences, thereby limiting direct comparisons among studies. Since hormones play multiple roles in metabolism, neuroimaging studies have suggested hypothalamic dysfunction, and clinical observation has noted weight changes across the disease course, meaning that further research is warranted. Despite limitations in methods, our meta‐analyses showed that cortisol showed significance across studies with increasing levels with disease progression prominent in mid‐stage HD after clinical motor diagnosis.

Significant differences across HD cohorts in one study detected extreme effect sizes in leptin from before (ie, preHD) to after clinical motor diagnosis and interpreted findings as support for autonomic dysfunction manifested in an absence of nocturnal dipping of arterial blood pressure. These findings are consistent with clinical observations of weight loss associated with manifest disease onset. Previous reviewers have cautioned against single‐measurement studies and encourage 24‐hour assessments with longitudinal follow‐up to determine how hormone changes may impact endocrine markers in HD.

In concert with endocrine markers, HD experts have investigated metabolic markers since the late 1960s because of early PET glucose studies^65^ and associations between levels of branched chain amino acids and clinical characteristics (ie, weight loss and rate of progression).^66^ Some studies have observed correlations of lipid and cholesterol metabolism with triplet repeat expansion and reductions of caudate volume.^67^, ^68^ A primary brain cholesterol metabolite reduction 24(s) hydroxycholesterol (24‐OHC) has been consistently observed in HD plasma; findings are supported by reduced levels of the cholesterol precursors lanosterol and lathosterol and of the bile acid precursor 27‐OHC. Given the crucial role of astrocytes in support of the CNS, maintenance of brain homeostasis may be reflected using these markers. Meta‐analyses were significant both for all HD versus NC in 24‐OHC as well as for early‐stage HD in both 24‐OHC and 27‐OHC. HDL was significant as a biomarker in mid‐stage HD or HD‐ISS Stage 3.

Metabolic markers for HD have been reported in many previous preclinical and human HD publications though consensus remains indefinable. While absolute levels are impossible to compare across studies, there are consistencies within and across studies. One comprehensive review purports that, although metabolic changes are not causal of HD pathogenesis, evidence of flux in responses to changing transcriptional disruptions and increased energy indicates that treatments may prolong the homeostatic compensation.^69^ Support for this premise derives from efforts to delay apoptosis in HD mice by assisting with sufficient energy production. Interventions to enhance energy production or upregulate metabolism have ameliorated disease phenotypes and extended lifespan in HD mouse models. A recent review in a special issue dedicated to HD concludes that metabolomics research is an essential tool to understand the systemic functional and pathophysiological pathways to identify early biomarkers with value for intervening in disease progression when cellular dysfunction has not progressed irreversibly.^70^ Based on the sheer number of significant findings reported, further research may be beneficial for metabolic markers. Larger‐scale collaborative efforts (and possibly measures with reduced variance) are likely needed to test potential biomarkers with rigor.

While clinical success rates for experimental therapeutics have been declining over the past few decades, they have remained stable at 5% in recent years.^71^ Despite recent approvals, the general trend continues to be low success rates, due to several factors: the rapidly evolving intricacy/complexity of the brain, unclear discordant data from experimental and clinical studies, poor clinical diagnostics, lack of functional endpoints, and high cost and complexity of techniques. The pathogenesis of HD is only partly understood, and further biomarker research has the potential to improve understanding of the underlying neurodegenerative pathway dysfunction(s), potentially leading to intervention targets. Given the complexity and potential heterogeneity of pathway dysfunction in HD, multiple treatments may perhaps be helpful at varying stages of the disease and dependent upon a specific case. For example, research suggests that the clinical relevance of specific biomarkers can vary widely across the disease course. Whereas microRNA differences in postmortem brain showed 16 highly significant differences between HD cases and controls, CSF samples from prodromal and early manifest HD showed significant differences in distinct microRNA levels very far from onset that plateaued before motor diagnosis of HD.^72^

Such findings support having a widespread, multivariate model of biomarker identification for clinical trials that may vary depending on disease stage. The National Academies' Forum on Neuroscience and Nervous System Disorders public workshop proposed an integrative approach where multimodal biomarkers could be derived from multiple measurements as is utilized in cholesterol measures (eg, HDL/low‐density lipoprotein [LDL]) and vital signs^73^ and as was most recently illustrated in Alzheimer's disease and mild cognitive impairment.^74^ Findings in this emerging field suggest that variability in biomarker performance is best when combining biomarkers, leading to improved clinical prediction models less affected by random error. Drug development for neurodegenerative diseases has a high risk of failure, and strategies to increase the probability of success with the prioritization of investments in projects that are most likely to succeed are needed. Given declining public trust and increased scrutiny of the FDA, NIH, and Centers for Disease Control and Prevention (CDC), rigor is critical to re‐establish the superiority of the scientific method over opinion, media frenzy, and popularity viewpoints from influencers.

The development and validation of biomarkers enable precision of drug development and facilitate a patient‐centered approach to therapeutics. As one of the primary foundations of new treatments, biomarkers must be elevated in importance and accuracy to be established as critical drug development tools within a specific context of use. The most significant finding from this review is, however, the lack of standards and compliance with professional reporting guidelines. Rigor and reporting for biomarker progress are particularly pertinent as the HD field continues to advance experimental therapeutics. Some of the most rudimentary research methods –demographics, study design, inclusion and exclusion criteria, data quality, analytic methods, reproducibility, definition of disease characteristics – are inconsistent across studies. Although the healthcare field showcases the dilemma of ethnicity, race, socioeconomic status, etc. and the influence that such variables have on the quality of knowledge obtained, the field of biomarker development remains focused on the output, rather than the input.^75^, ^76^ The ability to select clinically relevant biomarkers is dependent on the transparency and reproducibility of studies.

While we have long known of widespread publication bias in biomarker progress secondary to the lack of reporting non‐significant findings, the current literature is marked by foreseeable, identifiable, and reparable limitations. The primary variable limiting advancement across biomarker studies in HD is the inconsistent usage of study enrollments. It is impossible to compare across studies participants variously defined by CAG, CAG‐Age‐Product (CAP), Disease Burden Score (DBS), Normalized Prognostic Index score (PIN), and HD‐ISS.^25^, ^26^, ^77^ Documentation of stage‐specific biomarkers will require a fieldwide agreement on standards. BSQuAT‐HD ratings from two independent raters were consistent and only slightly better than those reported in the original publication of the measure.^20^ Though it is disappointing that the average score was just over half of the recommended checklist, we found a significant moderate positive correlation (n = 0.47) with study rating and year of publication, suggesting subtle improvements over time.^78^

Our findings are consistent with those reported in the revised STARD (Standards for Reporting of Diagnostic Accuracy Studies) statement: despite publication in two dozen international journals as well as endorsements by editorials, commentaries, and journal publication guidelines, performance metrics continue to be grim.^79^ It must be interrogated why hundreds of biomarker studies could be published in peer‐reviewed journals with fewer than half providing the fundamental information essential to advance clinical translational sciences. Since even the simplest biomarker study likely demands over 1000 human‐hours to conduct, contributors presumably desire significant impact for the time commitment. It is unlikely that there is purposeful failure to fully document research that is reproducible, so a call to action from all components of the scientific team may be appropriate.

Biomarker development and validation are vital to effective, efficient clinical trials. Efficiency concerns are heightened for rare disorders since there are fewer participants for clinical trials. We strongly suggest that journals, reviewers, and editors double down on sufficient data reporting using STARD.^79^ We further suggest that training programs for advanced degrees in the biomedical sciences mandate that students learn biomarker and clinical trial guidelines as part of the adjudication process to sustain scientific advancement. This review's findings may be critical to prepare the field for success in the development of new treatments for HD. In expectation of multimodal and machine learning advancements, we also suggest strict adherence to the Transparent Reporting of studies on prediction models for Individual Prognosis or Diagnosis (TRIPOD) most recently revised reporting recommendations to improve use of research time, effort, and money.^80^

## Author Roles

(1) Research Project: A. Design, B. Organization, C. Execution; (2) Statistical Analysis: A. Design, B. Execution, C. Review; (3) Manuscript Preparation: A. Drafting Initial Version, B. Editing Final Version.

J.S.P.: 1A, 1B, 2A, 2C, 3A, 3B.

N.A.B.: 1A, 1B, 1C, 3A, 3B.

J.D.C.: 1C, 3A, 3B.

A.W.: 3A, 3B.

A.M.K.: 1C, 3B.

M.L.J.: 1C, 3B.

A.P.: 1C, 2A, 2B, 3B.

D.K.B.: 1B, 1C, 3A, 3B.

H.Z.: 3B.

K.M.S.: 3B.

## Financial Disclosures of All Authors (for the Preceding 12 Months)

J.S.P.: Received support from research grants awarded by NIH/NINDS (U01NS103475; U01NS105509), NIH/NIA (RF1AG074608; R01AG082208; R01AG085602; R01AG082174), and DHHS/FDA (U01FD008399). N.A.B., J.D.C., A.W., A.M.K.: None. M.L.J.: Received support from the Ivy Dreizin Memorial Fund for continuing education awarded by the Department of Neurology, University of Wisconsin School of Medicine and Public Health. A.P., D.K.B.: None. H.Z.: Is a Wallenberg Scholar and a Distinguished Professor at the Swedish Research Council supported by grants from the Swedish Research Council (#2023‐00356, #2022‐01018, and #2019‐02397), the European Union's Horizon Europe Research and Innovation Programme under Grant Agreement No. 101053962, Swedish State Support for Clinical Research (#ALFGBG‐71320), the Alzheimer Drug Discovery Foundation (ADDF), USA (#201809‐2016862), the AD Strategic Fund and the Alzheimer's Association (#ADSF‐21‐831376‐C, #ADSF‐21‐831381‐C, #ADSF‐21‐831377‐C, and #ADSF‐24‐1284328‐C), the European Partnership on Metrology, co‐financed from the European Union's Horizon Europe Research and Innovation Programme and by the Participating States (NEuroBioStand, #22HLT07), the Bluefield Project, Cure Alzheimer's Fund, the Olav Thon Foundation, the Erling‐Persson Family Foundation, Familjen Rönströms Stiftelse, Stiftelsen för Gamla Tjänarinnor, Hjärnfonden, Sweden (#FO2022‐0270), the European Union's Horizon 2020 Research and Innovation Programme under the Marie Skłodowska‐Curie Grant Agreement No. 860197 (MIRIADE), the European Union Joint Programme–Neurodegenerative Disease Research (JPND2021‐00694), the NIHR University College London Hospitals Biomedical Research Centre, and the UK Dementia Research Institute at University College (UCL) (UKDRI‐1003); and has served on scientific advisory boards and/or as a consultant for AbbVie, Acumen, Alector, Alzinova, ALZpath, Amylyx, Annexon, Apellis, Artery Therapeutics, AZTherapies, Cognito Therapeutics, CogRx, Denali, Eisai, LabCorp, Merry Life, Nervgen, Novo Nordisk, Optoceutics, PassageBio, Pinteon Therapeutics, Prothena, Quanterix, Red Abbey Labs, reMYND, Roche, Samumed, Siemens Healthineers, Triplet Therapeutics, and Wave; has given lectures sponsored by Alzecure, BioArctic, Biogen, Cellectricon, Fujirebio, Lilly, Novo Nordisk, Roche, and WebMD; and is a co‐founder of Brain Biomarker Solutions in Gothenburg AB (BBS), which is a part of the GU Ventures Incubator Program (outside the submitted work). K.M.S.: Received clinical trial funding from Inhibikase, ASKBIO, and Aspen.

## Supporting information


**Table S1.** Reviewer coding categories for publication exclusion according to Silajdžić and Björkqvist.

**Table S2.** Summary of the 55 articles included in the present review.

## Data Availability

The data that supports the findings of this study are available in the supplementary material of this article.
