# Supplementary material for: Systematic Review with Meta‐Analysis of Biofluid Markers for Huntington's Disease
Source: Mov Disord. 2025 Oct 13;40(12):2578–95. doi: 10.1002/mds.70067 (PMC12710193; doi:10.1002/mds.70067)
Supplement: Supplementary file 1 — Table S1. Reviewer coding categories for publication exclusion according to Silajdžić and Björkqvist. [file MDS-40-2578-s001.docx]

Supplemental Table 1: Reviewer coding categories for publication exclusion according to Silajdžić and Björkqvist

| Reason Number | Rationale for exclusion |
| --- | --- |
| Reason 1 | Lacking necessary/relevant data (e.g., only presenting box plots, has mean but no standard deviation data, etc.). |
| Reason 2 | Does not relate to fluid biomarkers (e.g., studies that research DNA, RNA, microRNA, telomere length). |
| Reason 3 | Review and/or meta-analysis. |
| Reason 4 | Lacking necessary study populations (e.g., having no healthy controls, case reports, individuals with juvenile onset HD). |
| Reason 5 | Subjects are non-human models. |
| Reason 6 | Unrelated subject matter (e.g., does not relate to HD). |
| Reason 7 | Does not satisfy the coding scheme requirements from Silajdžić and Björkqvist’s 2018 review. |
