# Supplementary material for: Systematic Review with Meta‐Analysis of Biofluid Markers for Huntington's Disease
Source: Mov Disord. 2025 Oct 13;40(12):2578–95. doi: 10.1002/mds.70067 (PMC12710193; doi:10.1002/mds.70067)
Supplement: Supplementary file 2 — Table S2. Summary of the 55 articles included in the present review. [file MDS-40-2578-s002.docx]

**Supplemental Table 2:** Summary of the 55 articles included in the present review.

| **First Author (Year)** | **Control type**^1^ | **Control (n)** | **HD Mutation Carriers (n)** | **Study Design** |
| --- | --- | --- | --- | --- |
| Manyam et al. (1978) | 24–26-year-old men with no organic neurological disease | 5 | 5 | Cross-sectional |
| Oepen et al. (1982) | Non-choreic controls | 16 | 12 | Cross-sectional |
| Manyam & Tremblay (1984) | Normal controls | 15 | 8 | Cross-sectional |
| Manyam et al. (1990) | Normal controls | 9 | 6 | Cross-sectional |
| Perry & Hansen (1990) | Healthy controls | 7 | 14 | Cross-sectional |
| Bonilla et al. (1991) | Controls with similar environmental influences | 29 (Male)  57 (Female) | 7 (Male)  11 (Female) | Cross-sectional |
| Nicoli et al. (1993) | Controls suffering from headaches, discal herniation, non-inflammatory and non-degenerative CNS diseases | 12 | 11 | Cross-sectional |
| Reilmann et al. (1994)^3^ | Age- and sex-matched healthy controls | 21 (Plasma)  24 (Platelet) | 16 (Plasma)  28 (Platelet) | Cross-sectional |
| Andrich et al. (2004) | Healthy controls | 73 | 19 | Cross-sectional |
| Popovic et al. (2004) | Healthy controls | 20 | 15 | Cross-sectional |
| Lalić et al. (2008) | Controls matched by age, sex, and socioeconomic background | 22 | 29 | Cross-sectional |
| Leoni et al. (2008) | Partners/family members/volunteers with no major medical conditions | 67 | 33 (Pre-HD)  46 (Early HD)  20 (Mid-HD)  30 (Advanced HD) | Cross-sectional |
| Aziz et al. (2009a) | Age-, gender-, and body mass index (BMI)-matched controls | 9 | 9 | Cross-sectional |
| Aziz et al. (2009b) | Age-, gender-, and body mass index (BMI)-matched controls | 9 | 9 | Cross-sectional |
| Leoni et al. (2011) | Healthy age-matched controls | 134 | 48 (TFC Stage 1)  44 (TFC Stage 2)  35 (TFC Stages 3-5) | Cross-sectional |
| Túnez et al. (2011) | Healthy controls | 19 | 19 | Cross-sectional |
| Olsson et al. (2012) | Type not given | 18 | 48 | Cross-sectional |
| Leoni et al. (2013)^4^ | Mutation-negative controls | 30 | 21 (Low; Pre-HD)  47 (Medium; Pre-HD)  52 (High; Pre-HD) | Cross-sectional |
| Ciancarelli et al. (2014) | Healthy controls | 10 | 18 | Cross-sectional |
| Kalliolia et al. (2014) | Type not given | 15 | 27 | Cross-sectional |
| Chen et al. (2015) | Type not given | 23 | 14 | Longitudinal |
| Peña-Sánchez et al. (2015) | Age- and sex-matched controls | 29 | 14 | Cross-sectional |
| Politis et al. (2015) | Healthy age- and gender-matched controls | 12 | 12 | Cross-sectional |
| Süssmuth et al. (2015) | Healthy controls; partners of HD patients or volunteers | 29 | 37 | Cross-sectional |
| Nambron et al. (2016)^5, 6^ | Healthy controls | 12 (IEC)  14 (Reversed-phase HPLC)  15 (Hormonal and lipid metabolism markers) | 11 (IEC; Pre-HD)  14 (Reversed-phase HPLC; Pre-HD)  14 (Hormonal and lipid metabolism markers; Pre-HD)  12 (IEC and reversed-phase HLPC; Combined HD)  13 (Hormonal and lipid metabolism markers; Combined HD) | Cross-sectional |
| Byrne et al. (2017) | Healthy partners or siblings of *HTT* mutation carriers | 97 | 201 | Longitudinal and cross-sectional^2^ |
| Adamczak-Ratajczak et al. (2017) | Healthy age-matched, no history of medical illness | 10 | 11 | Cross-sectional |
| Moss et al. (2017)^7^ | Relatives of HD patients | 20 (Sample set 1)  23 (Sample set 2) | 14 (Sample set 1; Pre-HD)  23 (Sample set 2; Pre-HD)  14 (Sample set 1; Early HD)  18 (Sample set 2; Early HD)  8 (Sample set 1; Mid-HD)  13 (Sample set 2; Mid-HD) | Longitudinal and cross-sectional^2^ |
| Byrne et al. (2018) | Healthy controls | 20 | 60 | Longitudinal |
| Chang et al. (2018) | Normal controls | 47 | 22 | Cross-sectional |
| Diago et al. (2018) | Age- and gender-matched healthy controls | 23 (Pre-HD controls)  15 (Early HD controls) | 23 (Pre-HD)  15 (Early HD) | Cross-sectional |
| Niemelä et al. (2018) | Gene-negative controls | 18 (sCD27 analysis)  25 (all remaining reported biomarkers) | 11 (sCD27 analysis; Pre-HD)  13 (all remaining reported biomarkers; Pre-HD)  10 (sCD27 analysis; Early HD)  14 (all remaining reported biomarkers; Early HD) | Longitudinal and cross-sectional^2^ |
| Lamontagne-Proulx et al. (2019) | Healthy age- and sex-matched controls | 53 (EV CD41+/platelet, EV CD14+/monocyte, EV CD15+/granulocyte)  54 (all remaining reported biomarkers) | 10 (Pre-HD)  15 (Stage 1; Early HD)  12 (Stage 2; Early HD)  11 (Mid-HD)  10 (Stage 4; Advanced HD)  2 (Stage 5; Advanced HD) | Cross-sectional |
| Gregory et al. (2019) | Healthy age‐ and gender‐matched controls | 15 | 39 | Cross-sectional |
| Corey-Bloom et al. (2020) | Healthy controls | 22 (IL-6 and IL-1β; saliva)  44 (CRP; saliva)  27 (IL-6; plasma) | 24 (IL-6, IL-1β; saliva in Pre-HD)  35 (CRP; saliva in Pre-HD)  29 (IL-6; plasma in Pre-HD)  22 (IL-6; saliva in Early HD)  24 (IL-1β; saliva in Early HD)  33 (CRP; saliva in Early HD)  21 (IL-6; plasma in Early HD) | Cross-sectional |
| Rodrigues et al. (2020) | Healthy controls | 20 | 60 | Longitudinal and cross-sectional^2^ |
| Squadrone et al. (2020) | Healthy controls | 18 | 18 | Cross-sectional |
| Scahill et al. (2020) | Family history of HD but a negative genetic test; no known family history of HD | 51 (CSF-based analysis)  67 (plasma-based analysis) | 58 (CSF-based analysis)  63 (plasma-based analysis) | Cross-sectional |
| Ye et al. (2020) | Healthy controls | 24 | 27 | Cross-sectional |
| Cruickshank et al. (2021) | Healthy controls | 14 | 26 | Cross-sectional |
| Ou et al. (2021) | Age and gender-matched controls | 20 | 57 | Longitudinal and cross-sectional^2^ |
| Rodrigues et al. (2021) | Healthy controls | 18 (Anthranilic acid; plasma)  19 (3-Hydroxykynurenine and kynurenic acid; plasma)  20 (all remaining reported biomarkers) | 16 (Anthranilic acid; plasma in Pre-HD)  19 (Quinolinic acid and kynurenic acid; plasma in Pre-HD)  20 (all remaining reported biomarkers in Pre-HD) | Longitudinal |
| You et al. (2021) | Healthy age-matched; spouses and relatives of HD patients | 26 | 57 | Cross-sectional |
| Pfalzer et al. (2022) | Type not given | 12 | 16 | Cross-sectional |
| McColgan et al. (2022) | Gene-negative family members; individuals with no family history of HD | 67 | 64 | Cross-sectional |
| Lowe et al. (2022) | Healthy controls | 15 (Baseline collection)  11 (NfL and tau 24-month follow-up; CSF)  12 (NfL and tau 24-month follow-up; plasma) | 12 (mHTT 24-month follow-up; CSF in Pre-HD)  12 (NfL 24-month follow-up; plasma in Pre-HD)  13 (Tau 24-month follow-up; plasma in Pre-HD)  13 (NfL and tau 24-month follow-up; CSF in Pre-HD)  14 (GABA; CSF in Pre-HD)  15 (all remaining reported biomarkers in Pre-HD)  19 (GABA; CSF in Early HD)  22 (mHTT, NfL, and tau 24-month follow-up; CSF in Early HD)  23 (NfL and tau 24-month follow-up; plasma in Early HD)  26 (all remaining reported biomarkers in Early HD) | Longitudinal |
| Denis et al. (2023)^8^ | Age- and gender-matched healthy controls | 63 (WBC, lymphocytes, monocytes, and granulocyte analysis)  66 (all remaining reported biomarkers) | 15 (Pre-HD)  44 (WBC, lymphocytes, monocytes, and granulocyte analysis; Early HD)  48 (all remaining reported biomarkers; Early HD) | Cross-sectional |
| Chang et al. (2023) | Normal controls | 20 | 33 | Cross-sectional |
| Gray et al. (2024)^6, 7^ | Negative for CAG expansion or not genetically related family controls | 41 (25-OHC Year 3)  42 (25-OHC Year 1 and Year 4)  44 (all remaining reported biomarkers) | 46 (25-OHC Year 1; Pre-HD)  48 (25-OHC Year 3; Pre-HD)  50 (24(S)-OHC Year 1 and 25-OHC Year 4; Pre-HD)  51 (all remaining reported biomarkers; Pre-HD)  43 (25-OHC Year 1; Combined HD)  44 (25-OHC Year 3 and 27-OHC Year 3; Combined HD)  45 (all remaining reported biomarkers; Combined HD) | Longitudinal |
| Herrero-Lorenzo et al. (2024) | Caucasian healthy controls without the CAG mutation | 10 | 20 | Longitudinal |
| Korpela et al. (2024)^6, 9^ | Gene-negative and healthy controls | 8 (GFAP analysis)  19 (Aβ42 analysis) | 13 (Pre-HD)  26 (GFAP analysis; Combined HD)  27 (Aβ42 analysis; Combined HD) | Cross-sectional |
| Maiuri et al. (2024)^6,^ ^10^ | Healthy controls | 15 (Hemoglobin analysis)  17 (PAR analysis) | 17 (Hemoglobin analysis; Pre-HD)  19 (PAR analysis; Pre-HD) 31 (Hemoglobin analysis; Combined HD)  35 (PAR analysis; Combined HD) | Longitudinal |
| Pfalzer et al. (2024) | Individuals without a history of HD | 12 | 32 | Cross-sectional |
| Voysey et al. (2024)^6 ,7^ | Healthy controls | 6 (Baseline)  11 (Time 2)  8 (Time 3)  9 (Time 4) | 7 (Baseline; Pre-HD)  5 (Time 2; Pre-HD)  4 (Time 3; Pre-HD)  7 (Time 4; Pre-HD)  6 (Baseline; Combined HD)  13 (Time 2; Combined HD)  13 (Time 3; Combined HD)  8 (Time 4; Combined HD) | Longitudinal |
| Scahill et al. (2025) | Age-matched controls | 51 | 62 | Longitudinal and cross-sectional^2^ |

**Footnote:** A portion of the articles contained various population sizes across biomarker analysis

^1^ Wording used to describe the controls reflects the descriptions provided in the reviewed articles.

^2^Longitudinal and cross-sectional notation indicates a percentage of the cross-sectional cohort was chosen to undergo additional longitudinal analysis.

^3^Authors elected to assess biomarker levels within both platelets and plasma.

^4^Authors elected to separate Pre-HD population into low, medium, and high groups based on 24OHC concentration.

^5^IEC: Ion exchange chromatography

^6^Combined HD refers to all HD participant data presented as one cohort, with no mention of staging in the reviewed article.

^7^Wording of timepoints or populations (e.g., Sample set 2, Year 3, etc.) refer to the specific language used by authors in the reviewed article.

^8^WBC: White blood cells

^9^GFAP: Glial fibrillary acidic protein

^10^PAR: Poly adenosine diphosphate (ADP)-ribose
